# Longing for the Past and Longing for the Future: A Phenomenological Assessment of the Relation Between Temporal Focus and Readiness to Change Among People Living With Addiction

**DOI:** 10.3389/fpsyg.2020.01794

**Published:** 2020-07-21

**Authors:** Melissa M. Salmon, Michael J. A. Wohl

**Affiliations:** Department of Psychology, Carleton University, Ottawa, ON, Canada

**Keywords:** nostalgia, temporal focus, addiction, readiness to change, gambling

## Abstract

At present, the dominant motivational strategy to facilitate behavior change among those living with addiction is to focus one’s attention on the better possible future that may result from cutting down or cessation. However, research is now emerging that suggests nostalgic reverie (i.e., sentimental longing) for life lived before addiction can also motivate behavior change. In the current research, we explore the conditions in which longing for a better future free of addiction and longing for one’s past that was free of addition might motivate change. To this end, we assessed first-person experiential narratives of problem gamblers to better understand how they feel about their past or future without gambling, and how those feelings may relate to motivation to change. Problem gamblers were randomly assigned to either write about their lived past before gambling (*n* = 31) or their expected future without gambling (*n* = 26). Each narrative was systematically examined to identify recurrent themes and cluster these narratives according to similarly expressed themes. In the past condition, participants reported their life before gambling was either positive (Cluster P1) or difficult (Cluster P2). Gamblers with a positive past described how their life, character, close relationships, and the activities they engaged in before gambling were more meaningful. Importantly, these gamblers also reported feeling more nostalgic for life without gambling and were more ready to change their behavior than gamblers with a difficult past. In the future condition, participants were either positive (Cluster F1) or ambivalent (Cluster F2) about a future free from gambling. Gamblers who expected a positive future described how they expect their emotions, finances, and the activities they will engage in to be more positive without gambling. Compared to those ambivalent about their future, these gamblers also reported a future without addiction to be more vivid and had more desire to change their behavior, but there were no between-cluster differences in readiness to change. These findings demonstrate unique differences in how gamblers perceive their past and future without gambling, and shed a novel light on how each temporal focus might motivate behavior change among those living with addiction.

## Introduction

People living with addiction often do not behave in their best interest. Although they may objectively recognize their behavior is causing themselves (and others) harm (see Lesieur and Custer, 1984; Petry, 2005), the rate of behavior change is alarmingly low. This is because behavior change is difficult, as evidenced by the many people who fail to produce even a single change attempt (DiClemente et al., 1991), let alone take the necessary steps to successfully quit an addictive behavior (Miller and Rollnick, 2002). At present, the dominant motivational strategy to facilitate behavior change among those living with addiction is to facilitate longing for a better possible future that may result from cutting down or cessation (see Markus and Nurius, 1986; Ajzen, 1991; Oettingen, 2000). However, emerging research suggests that nostalgic reverie for life lived before the addiction took hold (i.e., sentimental longing for the past) has behavior change utility. In the current research, we explored the conditions under which longing for a post-addiction future and longing for the pre-addicted past may be most effective in motivating behavior change.

An individual’s ability to focus on the future has its benefits. Those who have a dispositional tendency to focus on the future typically have lower rates of engagement in a variety of addictive behaviors (see Keough et al., 1999), likely because future-focused people have a higher consideration for future consequences. Of course, there are future-focused people who live with addiction – a disposition that can facilitate behavior change. For example, smokers with a future focus were more likely to report having made a quit attempt within the subsequent 8 months (Hall et al., 2012) and were more likely to have quit smoking in the subsequent 4 years (Adams, 2009).

In light of the prophylactic and behavior change utility of a future focus, it is perhaps unsurprising that many traditional theories of behavior change focus on motivational strategies that attempt to promote the person to long for a better possible future without the addictive behavior in their repertoire. For example, Oettingen’s (2000) model of fantasy realization suggests fantasies about a desired future can be used to create strong commitment to a goal, which leads people to take action toward attaining that goal (Oettingen et al., 2001). Specifically, behavior change results from mental contrasting between the future and present. This is accomplished by first imagining a desired future (e.g., a future without addiction-related problems) and then reflecting on the negative reality that is impeding that future (e.g., financial loss due to excessive gambling). Pairing positive thoughts about the future with negative thoughts about current lived experiences makes both future and present simultaneously accessible (Kawada et al., 2004). This allows people to recognize the negative reality that is impeding them from realizing their desired future (Higgins and Chaires, 1980), thereby emphasizing a necessity to take action to overcome the present reality in order to attain the desired future (Oettingen, 2000).

Importantly, Oettingen’s model has been applied to addictive behaviors with some success. For example, Oettingen et al. (2010) showed that when smokers engaged in mental contrasting of a positive future without smoking with the negative reality of smoking, they took action to reduce their cigarette consumption. Importantly, this occurred only when participants had high expectations of success – those who had low expectations of success deferred behavior change. Moreover, Johannessen et al. (2012) found that dieting students were more likely to act in ways congruent with their diet goal (e.g., eating fewer high-calorie foods and more low-calorie foods, being more physically active) when they engaged in mental contrasting of a desired future with the negative reality. Taken together, these results provide some indication that looking forward to a desired future (i.e., without an addictive behavior) and contrasting it with the negative reality can lead people to take action toward quitting or cutting down on that behavior (Oettingen et al., 2010).

In a like manner, Markus and Nurius’ (1986) theory on possible selves argues that possible (future) selves function as incentives for behavior change. Specifically, people may be motivated to change their behavior by way of comparing the current self against a desired possible future self (which motivates approach behaviors) or a feared possible future self (which motivates avoidant behaviors; Markus and Nurius, 1986; Oyserman et al., 2004; vanDellen and Hoyle, 2008). Empirical evidence validates this supposition. Students who were presented with an image of a possible (future) exercising self significantly increased their exercise behavior in the subsequent 4 weeks, but only when they were also more oriented toward their future (i.e., higher tendency to consider future consequences; Ouellette et al., 2005). In a like manner, Hooker (1992) found that adults with a desired, health-related (future) possible self engaged in more health-related behaviors than did those who did not have a possible future self in the health domain. Indeed, lacking clear possible selves can also have consequences for behavior. As such, according to this understanding of behavior change, a lack of future orientation (i.e., when the future is vague) impedes behavior change.

Unfortunately, many people living with addiction may find it difficult to envisage a better future. For example, disordered gamblers have a skewed temporal orientation in that they tend to be present-focused (Toneatto, 1999; MacKillop et al., 2006), and fail to consider the future consequences associated with their betting decisions (Hodgins and Engel, 2002). Specifically, disordered gamblers tend to have significantly shorter time horizons in comparison to recreational gamblers and are less likely to predict events far into their future (Hodgins and Engel, 2002). These findings suggest that disordered gamblers may have difficulty planning for their future. Moreover, imagined future events among disordered gamblers typically lack detail and contextual information (Noël et al., 2017), which further undermines the planning process. As such, focusing gamblers toward a better possible future may not be the most effective means to motivate self-directed change.

If a better future is difficult for the gambler (in need of behavior change) to envision, there may be utility in focusing them on a positive past – one that was free of addiction. This is because many behavior change models recognize that the addictive behavior is negatively affecting the self (Markus and Nurius, 1986; Oettingen, 2000; Miller and Rollnick, 2002, 2012). Indeed, addiction often results in negative changes to people’s moods, behaviors, and self-esteem (Lesieur and Custer, 1984; Bergh and Kühlhorn, 1994). These changes can cause people to feel disconnected from who they were before the addiction took hold (i.e., they feel self-discontinuous). Although these feelings of self-discontinuity often represent a discontentment with the current self (Davis, 1979; Sedikides et al., 2008), the recognition that one’s quality of life was better before the addiction-related problems took hold is pivotal to understanding that behavior change is in one’s best interest.

For instance, Kim et al. (2017) found that feelings of self-discontinuity were associated with a greater likelihood of having attempted self-directed change over time, even when controlling for known barriers to change (e.g., shame, guilt, self-stigma). Nuske and Hing (2013) also reported that some disordered gamblers are motivated to engage in self-directed change after contrasting the positive past against the negative present reality of living with addiction. Although self-discontinuity, by definition, compares and contrasts the current self with the past self (Davis, 1979; Sani, 2008), mention of the past self is typically absent from many behavior change modalities (see Salmon et al., 2017). Given that self-discontinuity is associated with positive behavior change among people living with addiction, looking back to a more positive past (before the addiction took hold) may be an untapped avenue for self-directed change.

The clinical literature provides some clues about why a focus on a positive past may motivate change. Miller and Rollnick (2002) argued that in addition to focusing on the future, behavior change can be facilitated by encouraging reflection on the past. This technique involves motivating the client (e.g., a disordered gambler) to remember their life before problems with their addictive behavior emerged and contrasting those memories with how their life is now. According to Rosengren (2009), this process helps the person living with addiction re-establish values and reaffirm goals for the future. Given that disordered gamblers have difficulty planning for their future (Noël et al., 2017) and fail to consider the future consequences of their immediate actions (Hodgins and Engel, 2002), it would stand to reason that looking back to a more positive life lived *before* problems arose due to their gambling may be a viable means to motivate self-directed change. To the point, self-directed change may be a product of thoughts about how one’s addictive behavior has worsened with time, coupled with a longing to regain what was lost (e.g., values) as a result of addiction.

In fact, longing to return to a more positive past is a natural response to negative self-change (Davis, 1979; Best and Nelson, 1985; Sedikides et al., 2015). Put another way, feeling that the self has fundamentally changed for the worse (i.e., feeling self-discontinuous) elicits nostalgic reverie (i.e., a sentimental longing) for a more favorable past (Sedikides et al., 2015). Indeed, nostalgia is colloquially understood to be a positive emotional response to thoughts of days gone by. Nostalgia places the past in a good, idealistic light, which is contrasted against the stress of the life currently lived (Davis, 1979). Importantly, nostalgia helps people to regain a sense of meaning in life (Routledge et al., 2011) and increases optimism for the future (Cheung et al., 2013) by re-establishing a sense of continuity (Sedikides et al., 2015). In other words, although nostalgia initially stems from the perception that the current self is worse off than the past self, engaging in nostalgic reflection can help people feel closer to their favorable past self. This, in turn, promotes a regained sense of self-continuity (Sedikides et al., 2015).

One mechanism by which nostalgia re-establishes a sense of self-continuity is by fostering social connectedness (Sedikides et al., 2016). That is, nostalgia can boost perceived social support (Wildschut et al., 2006, 2010), counteract feelings of loneliness (Zhou et al., 2008), and promote prosocial behaviors (Stephan et al., 2014; Sedikides and Wildschut, 2016). In doing so, the important relationships with close others held in nostalgic memories are brought to the fore, which helps people to feel reconnected with all aspects of their past self (Sedikides et al., 2016). For this reason, nostalgia has been framed as an active coping resource (Sedikides et al., 2009) that motivates action to positively address life stressors (Stephan et al., 2014). Put another way, nostalgia is functional (Stephan et al., 2014; Abeyta et al., 2015; Sedikides and Wildschut, 2016), which may manifest among people living with addiction as motivation to return to their life lived before they began engaging in the addictive behavior.

As noted by Berg and Miller (1992), disordered gamblers often refer to their past nostalgically when asked to describe a future absent of addiction. This may be because nostalgia draws the person living with addiction closer to the more favorable past that existed before the development of problems associated with the addictive behavior. Because nostalgia restores a sense of self-continuity among people who feel that there has been fundamental self-change (Sedikides et al., 2015), nostalgic reverie for the pre-addicted self should motivate self-directed behavior targeted at recapturing the longed-for past – a past without the target behavior in their repertoire.

Providing empirical support for the self-directed change utility of nostalgia, Kim and Wohl (2015) found that among problem gamblers, as well as problem drinkers, a sense self-discontinuity (measured and manipulated) heightened nostalgic reverie for the pre-addicted self. Importantly, nostalgia for the pre-addicted self was positively associated with readiness to change. More recently, Wohl et al. (2018) demonstrated that experimentally induced nostalgia (stemming from self-discontinuity) motivated disordered gamblers, as well as problem drinkers, to make a quit attempt (relative to those in a control condition). Thus, focusing people on a point in their personal past when they were free of addiction may prove beneficial. That said, Salmon et al. (2018) observed that the power of nostalgizing for the pre-addicted self was restricted to people who believed that change was possible (i.e., they had incremental beliefs about the malleability of human behavior; see Dweck, 2008). As such, just as there are limits and boundaries to the motivating properties of a future focus (e.g., when the future is vague), there are likely contexts in which a past focus fails to effectively motivate change. The purpose of the research reported herein was to shed some light on the conditions in which a future and past focus may be beneficial in motivating behavior change among people living with addiction.

### Overview

In the current research, we recruited a sample of community problem gamblers who are not seeking treatment for their gambling-related problems to explore their lived past experiences before their gambling became problematic, as well as their anticipated future experiences once their gambling is no longer problematic. The aim of this qualitative exploration was to determine whether specific anchors exist within the past and future that may serve as motivation to take action to quit or cut down on problematic gambling behavior. To this end, we sought to classify various categories of lived and anticipated experiences that are associated with the extent to which gamblers are ready for and desire change. To test this idea, we used a numerically aided phenomenology approach (NAP; see Kuiken and Miall, 2001) – a procedure that allows for an assessment of different kinds of lived experiences within a set of qualitative narratives. This is accomplished via comparative reading of each narrative, which allows the researcher to identify recurring expressions and then paraphrasing those expressions to create categories of shared meanings.

By way of the NAP approach, we explored how the various meanings gamblers assign to their lived pasts and anticipated futures group together to form clusters according to the similarities in their profiles of meaning expressions. Focus was placed on the similarities and differences between the various types of lived experiences gamblers shared before their gambling became problematic. Whereas some gamblers may perceive their past before gambling as a generally positive time that they long to return to (i.e., they feel nostalgic for this time in their life), other gamblers may perceive the past as a place of pain that they are trying to avoid. Additionally, we examined the similarities and differences between gamblers’ outlooks toward their future. Whereas some gamblers may look forward to a future free of gambling, others may experience anxiety when tasked with envisioning a future without gambling. After providing their narratives, participants completed a questionnaire that further assessed their perceptions of and longing for their past and future. This questionnaire helped facilitate a more complete understanding of the meaning assigned to each temporal orientation. Specifically, participants completed measures that assessed, among other things, the vividness of the past and future, their longing for each temporal dimension, and the extent to which they were ready for and desired behavior change.

## Materials and Methods

### Participants

Participants were recruited from Amazon.com’s Mechanical Turk (MTurk). MTurk allows “workers” to complete small tasks for monetary compensation. Buhrmester et al. (2011) found that the majority of “workers” participate out of interest or to pass the time, rather than for the sake of compensation, making these participants a good source of data. Importantly, MTurk provides a reliable and diverse participant pool that behaves in ways consistent with known effects in psychology (Crump et al., 2013), and has been shown to be a reliable and valid means to recruit gamblers, drinkers, and cannabis users (Kim and Hodgins, 2017).

Participation on MTurk was limited to those who (1) were residents of the United States, (2) have spent at least $100 on gambling activities (e.g., slot machines, poker, roulette, sports betting) in the past 12 months, (3) think they have problems with their gambling (e.g., spend too much time or money gambling), and (4) were not in treatment for their gambling. Based on this eligibility criteria, we recruited 60 community problem gamblers (33 male, 27 female) who were not seeking treatment for their gambling problems. Participants’ age ranged from 21 to 73 years (*M* = 34.05, *SD* = 9.90).

The sample size was determined based on the recommendations of Kuiken and Miall (2001) to have at least 20 participants per hypothesized theme. Because two general themes (past focus and future focus) were to be examined, a sample size of 40 participants was determined to be appropriate. We added 10 participants to each theme (*N* = 60) to account for any poor data quality (e.g., insufficient or unclear responses).

All participants received US $3.00 for completing the study (approximately 30 min in duration). However, because the purpose of this study was to examine how problem gamblers think and feel about their past before gambling as well as their possible future without gambling, the sample used for analysis was further limited to only participants who exhibited moderate to disordered gambling severity. From the original sample of 60 participants, one participant was categorized as a low-risk gambler using the Problem Gambling Severity Index (PGSI; Ferris and Wynne, 2001), and thus was excluded from the analyses. Furthermore, two participants were also excluded from the subsequent analyses due to insufficient responses (i.e., they did not follow the writing prompts). Thus, the final sample consisted of 57 moderate and disordered gamblers (31 male, 26 female), ranging in age from 21 to 73 years (*M* = 34.09, *SD* = 10.05).

### Procedure and Measured Variables

A recruitment notice was posted on MTurk advertising the study as an opportunity for people to tell their story about their problems with gambling. Interested participants provided their informed consent and were assessed on their eligibility. Only participants who (1) were residents of the United States, (2) had spent at least $100 on gambling activities (e.g., slot machines, poker, roulette, sports betting) in the past 12 months, (3) thought they have problems with their gambling (e.g., spend too much time or money gambling), and (4) were not in treatment for their gambling continued to the full survey. Participants first reported their demographics (e.g., age and gender) as well as general information about their gambling behavior (e.g., time and money spent gambling). We then presented participants with a brief preface asking them to “please read the instructions carefully and provide honest responses” before randomly assigning participants to either a past focus or a future focus condition in which they completed a series of writing tasks.

In the past focus condition, participants were asked to “take some time to think about what your life was like before your gambling became problematic” and spend the next 10 min writing about this past. To increase the breadth of responses, additional prompts were included (i.e., “What filled your days? What were your relationships with others like? What were you like?”). A timer counting up was included on this survey page so that participants could keep track of how long they were writing for. After submitting their response, participants were then given the opportunity to add more to their story should they choose to.

Participants in the past focus condition were then presented with a series of face-valid items assessing various emotions and outcomes associated with their life before problem gambling. Specifically, participants responded to items assessing the clarity and vagueness of their past (i.e., “The life I lived before my gambling became problematic is vivid (i.e., clear) in my mind,” “The life I lived before my gambling became problematic is vague (i.e., fuzzy) in my mind”), longing (i.e., “I long for the life I lived without problematic gambling”), positive and negative emotions (e.g., “When gambling wasn’t problematic, I felt safe and secure in my life,” “It makes me feel anxious to think about the life I lived without gambling”), a sense of meaning (i.e., “The life I lived before I started gambling problematically was full of meaning”) and social connectedness (i.e., “Before my gambling became problematic, I felt more love in my life”). All items were anchored at 1 (*strongly disagree*) and 7 (*strongly agree*).

In the future focus condition, participants were presented with a similar writing prompt that was tailored toward a possible future without gambling. Specifically, they were asked to “take some time to think about what your life would look like if you decided to change your problematic gambling” and spend the next 10 min writing about this future. To increase the breadth of responses, additional prompts were included (i.e., “What would fill your days? What would your relationships with others be like? What would you be like?”). A timer counting up was included so that participants could keep track of how long they were writing for. After submitting their response, participants were then given the opportunity to add more to their story should they choose to.

Participants in the future focus condition were then presented with a similar series of face-valid items assessing various emotions and outcomes associated with their future life without gambling. Specifically, participants responded to items assessing the clarity and vagueness of their future [i.e., “The life I would live after my gambling is no longer problematic is vivid (i.e., clear) in my mind,” “The life I would live after my gambling is no longer problematic is vague (i.e., fuzzy) in my mind”], longing (i.e., “I long for the life I would live without problematic gambling”), positive and negative emotions (e.g., “When my gambling is no longer problematic, I will feel safe and secure in my life,” “It makes me feel anxious to think about the life I would live without gambling”), a sense of meaning (i.e., “The life I would live after I stop gambling problematically will be full of meaning”) and social connectedness (i.e., “After my gambling is no longer problematic, I will feel more love in my life”). All items were anchored at 1 (*strongly disagree*) and 7 (*strongly agree*).

All participants then completed Biener and Abrams’ (1991) single-item pictorial contemplation ladder, adapted for gambling behavior. Though the contemplation ladder was originally developed to assess readiness to quit smoking, it has been shown to be a strong measure of gamblers’ readiness to change (Hodgins, 2001; Kim and Wohl, 2015). The contemplation ladder is continuous and is anchored at 0 (*no thought of changing*) and 10 (*taking action to change – e.g., cutting down, enrolling in a program*). A score of 0–3 corresponds with DiClemente et al. (1991) pre-contemplation stage of change (i.e., not thinking about change), a score of 4–6 corresponds with the contemplation stage (i.e., thinking about change), a score of 7 or 8 corresponds with the preparation stage of change (i.e., preparing to change within the next 30 days), and a score of 9 or 10 is indicative of the action and maintenance stages, respectively (i.e., actively modifying unhealthy behavior). Following the contemplation ladder, participants also expressed their desire to change their gambling on a scale from 0 (*no desire*) to 9 (*full desire*). Participants were then asked whether they had previously made a quit attempt. This item was: “Have you ever made an attempt to quit or cut down on your gambling?” Responses to this item were dichotomous (*yes* or *no*).

Lastly, participants completed the PGSI (Ferris and Wynne, 2001). The PGSI is a continuous nine-item measure (α = 0.85) that assesses disordered gambling behavior (e.g., “Have you needed to gamble with larger amounts of money to get the same feeling of excitement?”) and the consequences of disordered gambling (e.g., “Have you felt guilty about the way you gamble or what happens when you gamble?”). Responses were anchored at 0 (*never*) and 3 (*almost always*). Participants’ scores were summed to obtain a total score (ranging from 0 to 27), which was used to classify participants into one of four categories. A gambler with a score of 0 was categorized as a non-problem gambler, 1–2 as a low-risk gambler, 3–7 as a moderate-risk gambler, and 8 or more as a disordered gambler. Participants were then directed to the debriefing page where the full nature of the study was disclosed.

For exploratory purposes, participants were also asked to either list the things they longed for most when thinking about their life before gambling became problematic (in the past condition), or to list the things they longed for most when thinking about what their life would look like if they decided to change their problematic gambling. Participants were encouraged to list as many things as they can think of in no particular order.

This research was reviewed and cleared by the Carleton University Research Ethics Board-B (CUREB-B).

## Results

A summary of demographics and self-reported gambling behavior in each condition can be found in Table 1.

**TABLE 1:** Demographics among participants in each condition.

	Past		Future		Total	
	*n*	%	*n*	%	*n*	%
Age^1^	35.65	(11.08)	32.23	(8.51)	34.09	(10.05)
**Gender**						
Male	16	51.6%	15	57.7%	31	54.4%
Female	15	48.4%	11	42.3%	26	45.6%
**PGSI category**						
Moderate-risk gambler	5	16.1%	3	11.5%	8	14.0%
Disordered gambler	26	83.9%	23	88.5%	49	89.0%
**Gambling frequency**						
More than once every 3 months	2	6.5%	0	0.0%	2	3.5%
More than once a month	7	22.6%	8	30.8%	15	36.3%
More than once a week	18	58.1%	14	53.8%	32	56.1%
More than once a day	4	12.9%	4	15.4%	8	14.0%
**Previous change attempt**						
Yes	21	67.7%	12	46.2%	33	57.9%
No	10	32.3%	14	53.8%	24	42.1%

*No significant differences between conditions on demographic items (*p* > 0.05). ^1^Mean age and standard deviation reported.*

### Analysis of the Experiential Narratives

Participants’ experiential narratives were systematically compared by the authors and two research assistants to identify similarly expressed meanings (see Kuiken and Miall, 2001 for a detailed description of these procedures). When sentences with similar meaning occurred in three or more narratives, they were paraphrased to reflect as much of their common meaning as possible. For example, the following statements from three different narratives were understood to express a common meaning: (1) “I was a person full of life,” (2) “I was a very sweet person,” and (3) “I was actually a real person.” The meaning that these statements had in common was paraphrased to reflect as much of their shared meaning as possible: “I was a better person before my gambling became problematic.” The wording of such paraphrases, called constituents, was established by making strict comparisons between similar meanings shared by these expressions within the set of narratives.

When a constituent was identified, each narrative within the dataset was systematically reread to determine whether the expressed meaning was present or absent. Through repeated readings, an array of 12 such constituents were identified for the past condition, and 12 constituents were identified for the future condition. Each of the constituents identified were neither rare (i.e., found in less than 10% of the narratives) nor ubiquitous (i.e., found in more than 90% of the narratives). The resulting arrays of constituents by participants for both conditions were subjected to an increase in sum of squares (Ward’s) hierarchical cluster analysis (using squared Euclidian distance coefficients). For the past condition, the cluster analysis on the 12 × 31 array revealed two distinct clusters of experiential narratives of one’s past before gambling. For the future condition, the same hierarchical cluster analysis on the 12 × 26 array also revealed two distinct clusters of experiential narratives of one’s anticipated future without gambling.

In both the past and future conditions, the prevalence of each constituent across clusters was compared to identify the constituents that differentiated one cluster from the other. A constituent was determined as differentiating if (1) it occurred in at least three members of the cluster; (2) it occurred at least twice as often as in the other cluster; and (3) the proportion of individuals expressing it within a cluster was greater than the proportion expressing it in the other cluster using the chi-square statistic (*p* < 0.05) as criterion. As clustering techniques maximize between-cluster differences, it should be noted that the chi-square statistic was only used descriptively to determine significant differences in the proportion of constituent expressions rather than for testing significant departures from group equivalence (Everitt et al., 2004). For a more detailed account of the analyses, please see OSF^1^.

### Past Condition

The characteristic attributes of each cluster, along with the non-differentiating characteristics, are summarized in Table 2. Example excerpts from narratives whose profiles most nearly resembled the ideal type for each cluster are also presented in the summary descriptions that follow.

**TABLE 2:** Proportion of cluster members expressing each constituent in each of the two clusters in the past condition.

	Cluster	
Constituent	P1	P2
C1. My life was more positive before gambling became problematic	0.75*	0.00
C2. I had better social connections before my gambling became problematic	0.95*	0.18
C3. I was involved in more meaningful activities before my gambling became problematic	0.80*	0.27
C4. I was a better person before my gambling became problematic	0.45*	0.09
C5. Gambling took over my life (themes of discontinuity)	0.30*	0.00
C6. Gambling has not changed the quality of my life (or my social connections)	0.00	0.73*
C7. Parts of my past were positive and parts of my past were negative (mixed bag)	0.00	0.36*
C8. There was a pivotal (traumatic) event that triggered my gambling	0.00	0.27*
C9. I will not change my gambling behavior	0.00	0.45*
C10. I was happier before my gambling became problematic	0.35	0.09
C11. My financial situation was better before my gambling became problematic	0.60	0.27
C12. I am resistant to changing my gambling behavior	0.25	0.09

*More frequently present than in the other cluster, *p* < 0.05.

#### Cluster P1

Participants in the first past cluster (*n* = 20) indicated a major shift between their past before gambling became problematic and their life now (Constituent 5), suggesting that the presence of gambling became overwhelming (e.g., “Gambling took over my life”; “Gambling changed my life completely”). Perhaps as a result of this felt discontinuity between past and present, people perceived that their life before gambling was generally more positive (Constituent 1; e.g., “Life was calmer”; “I used to enjoy life a lot more”), suggesting that gambling has changed their life for the worse (e.g., “Everything has become harder for me”; “I had fewer problems”). Within this positive (pre-problematic gambling) past, almost everyone mentioned specifically that they were more socially connected before their gambling became problematic (Constituent 2). These social connections referred to time spent with family and friends (e.g., “I played sports with friends”; “Quality family times which ought to be spent together are not as numbered as before”), having more open and trusting relationships (e.g., “My husband and kids could trust me”; “I was unselfish and generous to my friends”), or having established relationships that have since declined or have been lost altogether (e.g., “I was married to the love of my life and could not have been happier”; “My relationship with my family was much more stable”). Moreover, participants also engaged in more positive and meaningful activities (that were not gambling) in their past (Constituent 3), which largely consisted of hobbies and other recreational activities (e.g., “I liked to go fishing and hunting”; “I spent more time doing leisure activities instead of figuring out everything I wanted to do to gamble”). In addition to enjoying a better quality of life, participants in Cluster P1 also described themselves as being a better person before they began gambling problematically (Constituent 4). These judgments of character often comprised their own disposition (e.g., “I was a person full of life”; “I was a very sweet person”) and their values (e.g., “I was a pretty transparent, honest person”). In sum, participants in this cluster reported a significant change (for the worse) in the quality of their life after gambling became problematic, and wrote about their past fondly, claiming specific aspects of their life and their character as being more favorable.

#### Cluster P2

Participants in the second past cluster (*n* = 11) painted a less positive picture of their past. Their comments suggested that life was already quite hard before they started gambling problematically (Constituent 7), referring to both the difficult times they faced (e.g., “I seldom found myself outside of the house with little friends”) and their general dissatisfaction with life (e.g., “Before it got bad I was so bored with life”). These difficult times may have triggered the onset of their problem gambling (Constituent 8), with traumatic moments typically involving the death of a loved one (e.g., “In the year before I started, two of my siblings committed suicide”; “What broke the camel’s back was when I lost my uncle”). Likely because participants reported that their life was already quite difficult before their gambling became problematic, they also mentioned that the quality of their life did not change once they started gambling problematically (Constituent 6). That is, participants didn’t see gambling as having made their life any worse than it already was [e.g., “My life was (and still is) quite uninteresting”; “My life was already very hard before I started gambling”]. Rather, some people see their gambling as having added something to their hard life, expressing that they are unwilling to change their gambling behavior (Constituent 9). These people often framed gambling in a positive light while ignoring the potential harms (e.g., “It’s a rush I can’t describe”; “Gambling really just creates a little bit of excitement”) or provided their motive for continued play (e.g., “I am trying to win enough money to leave this horribly boring area”; “They say you cannot win if you do not play”). In sum, participants in this cluster described a past that was already quite difficult to begin with, and that gambling may have added an element of excitement to this difficult life.

#### Past Perception Ratings

To compare the two clusters further, a series of one-way ANOVAs were conducted on seven face-valid items assessing participants’ perceptions of their past before problem gambling. As indicated in Table 3, there were no significant differences between clusters on how vivid or vague their past before problematic gambling was in their mind. There were also no cluster differences in participants’ ratings of how anxious it makes them feel to think about the life they lived without gambling. However, participants in Cluster P1 (*M* = 5.60, *SD* = 1.23) expressed greater longing for their past than those in Cluster P2 (*M* = 3.27, *SD* = 1.90), *F*(1,29) = 17.15, *p* < 0.001. Moreover, participants in Cluster P1 (*M* = 5.95, *SD* = 1.00) also reported that they felt significantly more safe and secure in their life before gambling than did those in Cluster P2 (*M* = 3.55, *SD* = 1.86), *F*(1,29) = 22.17, *p* < 0.001. Participants in Cluster P1 (*M* = 5.65, *SD* = 1.04) also reported that their life before gambling became problematic was more full of meaning that did participants in Cluster P2 (*M* = 3.27, *SD* = 1.90), *F*(1,29) = 20.50, *p* < 0.001. Lastly, participants in Cluster P1 (*M* = 5.80, *SD* = 1.06) indicated that they also felt significantly more love in their life than did participants in Cluster P2 (*M* = 3.18, *SD* = 1.78), *F*(1,29) = 26.70, *p* < 0.001.

**TABLE 3:** Past perception ratings and behavior change measures between past clusters.

	Cluster P1		Cluster P2			
Item	*M*	*SD*	*M*	*SD*	*F*	*p*
The life I lived before my gambling became problematic is vivid in my mind	5.55	1.23	5.09	2.12	0.59	0.45
The life I lived before my gambling became problematic is vague in my mind	2.55	1.64	2.73	1.85	0.08	0.79
I long for the life I lived without problematic gambling	5.60	1.23	3.27	1.90	17.15	<0.001
When gambling wasn’t problematic, I felt safe and secure in my life	5.95	1.00	3.55	1.86	22.17	<0.001
It makes me feel anxious to think about the life I lived without gambling	4.10	2.02	3.73	2.33	0.22	0.65
The life I lived before I started gambling problematically was full of meaning	5.65	1.04	3.27	1.90	20.50	<0.001
Before my gambling became problematic, I felt more love in my life	5.80	1.06	3.18	1.78	26.70	<0.001
Readiness to Change	7.15	1.60	4.64	2.50	11.71	0.002
Desire to Change	6.85	1.66	5.00	2.76	5.48	0.03

#### Behavior Change Measures

All participants were asked to rate the extent to which they felt ready to change their gambling behavior, in addition to the extent to which they desired change. Participants were also asked whether they had made a previous attempt to change their gambling behavior. Results indicated that participants in Cluster P1 (*M* = 7.15, *SD* = 1.60) reported a significantly greater readiness to change their gambling behavior than did participants in Cluster P2 (*M* = 4.64, *SD* = 2.50), *F*(1,29) = 11.71, *p* = 0.002. Participants in Cluster P1 (*M* = 6.85, *SD* = 1.66) also reported a significantly greater desire to change their gambling behavior than did participants in Cluster P2 (*M* = 5.00, *SD* = 2.76), *F*(1,29) = 5.48, *p* = 0.03. However, there were no cluster differences in the likelihood a previous attempt to change their gambling behavior had been made, *X*^2^(1) = 0.13, *p* = 0.72.

### Future Condition

The characteristic attributes of each cluster, along with the non-differentiating characteristics, are summarized in Table 4. Excerpts from narratives whose profiles most nearly resembled the ideal type for each cluster are also presented in the summary descriptions that follow.

**TABLE 4:** Proportion of cluster members expressing each constituent in each of the two clusters in the future condition.

	Cluster	
Constituent	F1	F2
C1. I will be happier when my gambling is no longer problematic	0.73*	0.07
C2. My finances will be better after my gambling is no longer problematic	1.00*	0.33
C3. I will engage in more meaningful activities after my gambling is no longer problematic	1.00*	0.47
C4. Parts of my life will be better, parts of my life will stay the same, and parts of my life will be worse (mixed bag)	0.00	0.73*
C5. I am resistant to changing my gambling behavior	0.27	0.67*
C6. My social connections will be better after my gambling is no longer problematic	0.91	0.60
C7. My life will be more positive when my gambling is no longer problematic	0.55	0.20
C8. I will be a better person when my gambling is no longer problematic	0.46	0.13
C9. Mention of a new beginning	0.55	0.27
C10. I will not change my gambling behavior	0.00	0.27
C11. My future without gambling is vague/uncertain	0.09	0.20
C12. My life would have been hypothetically better if I hadn’t gambled (expression of upward counterfactual)	0.09	0.13

*More frequently present than in the other cluster, *p* < 0.05.

#### Cluster F1

Participants in the first future cluster (*n* = 11) anticipated that their future will be more positive than their current situation. Within this positive future, participants reported that they expect to experience more positive emotions when their gambling is no longer problematic (Constituent 1). That is, participants reported that they will be happier (e.g., “I think I would be much happier”; “I will feel much more calm”) and avoid negative emotions, such as anxiety (e.g., “I will be patient and avoid anxiety”; “My emotions will be much more stable”). Participants also described specific aspects of their life that they anticipate as being more favorable when their gambling is no longer problematic, such as their financial situation (Constituent 2). While suggesting that they would have more money in general (e.g., “I’d definitely have a lot of disposable income”; “I would have more money saved”), participants also mentioned that they can divert the money they spend on gambling into other productive areas, such as investment (e.g., “The amount being spent on gambling can be saved for a much more better form of investment”; “I could invest that money in retirement”). They also reported that they plan on engaging in more meaningful activities when their gambling is no longer a concern (Constituent 3), such as pursuing hobbies and travel (e.g., “I would have more time to read, cook, live my life”; “I would want to go on vacation and visit places”) or engaging in more productive activities [e.g., “I will…do things that are positive to (myself) like readings books and journals, doing some exercise”; “I will have much more time focusing on immediate and future goals”]. In sum, participants in this cluster reported being optimistic about a future in which they will have more money, engage in more positive activities, and by doing so, be happier in life.

#### Cluster F2

Participants in the second future cluster (*n* = 15) described a future with more ambivalence (Constituent 4). That is, while quitting gambling itself was described as being a positive change, they anticipated that other aspects of their life will either stay the same [e.g., “I don’t know exactly how much would change”; “My life would pretty much (stay) the same”] or worsen (e.g., “I think I would feel like something is missing in my life…I’m worried that I would feel bored all the time”; “I would be a bit less fun and less driven”). This ambivalence extended toward thoughts of changing their gambling behavior, with participants expressing a resistance to change (Constituent 5). Though participants acknowledged that change was in their best interest, participants largely commented on how difficult change will be (e.g., “My life would be a lot better if I change my gambling, but I don’t think I can stop”; “I think it would be very hard…I wish I could quit”) as well as how frustrated they were with their current situation (e.g., “It is frustrating and I feel like a failure in life, but there’s not much I can do”; “It’s not like I haven’t thought about this before or tried stopping”). In sum, while participants in this cluster envisioned a future where changing their gambling behavior will be rewarding, they also understand that hardships will arise when overcoming their current situation.

#### Future Perception Ratings

To compare the two clusters further, a series of one-way ANOVAs were conducted on seven face-valid items assessing participants’ perceptions of their future when their gambling is no longer problematic. These face-valid items are identical to the past perception items, but were adapted for the future. As indicated in Table 5, there were no significant differences between clusters in participants’ ratings of how anxious it makes them feel to think about the life they will live without gambling. However, participants in Cluster F1 (*M* = 6.00, *SD* = 0.78) reported that the life they would live after their gambling is no longer problematic is significantly more vivid in their mind than did those in Cluster F2 (*M* = 4.47, *SD* = 1.46), *F*(1,24) = 10.02, *p* = 0.004. Not surprisingly, participants in Cluster F2 (*M* = 4.27, *SD* = 2.09) in turn reported that the life they would live without gambling is significantly more vague in their mind than did participants in Cluster F1 (*M* = 2.45, *SD* = 1.67), *F*(1,24) = 5.58, *p* = 0.03. Moreover, participants in Cluster F1 (*M* = 6.09, *SD* = 1.22) expressed greater longing for the life they would live without gambling than did those in Cluster F2 (*M* = 4.47, *SD* = 1.69), *F*(1,24) = 7.35, *p* = 0.01. Participants in Cluster F1 (*M* = 6.09, *SD* = 0.83) also reported that they will feel significantly more safe and secure in their life without gambling than will participants in Cluster F2 (*M* = 4.67, *SD* = 1.72), *F*(1,24) = 6.40, *p* = 0.02. Participants in Cluster F1 (*M* = 6.27, *SD* = 0.91) also reported that their life without gambling will be significantly more full of meaning than did participants in Cluster F2 (*M* = 5.33, *SD* = 1.18), *F*(1,24) = 4.89, *p* = 0.04. Lastly, participants in Cluster F1 (*M* = 5.82, *SD* = 1.25) indicated that they will feel more love in their life than will participants in Cluster F2 (*M* = 4.53, *SD* = 1.73), *F*(1,24) = 4.38, *p* = 0.05.

**TABLE 5:** Future perception ratings and behavior change measures between future clusters.

	Cluster F1		Cluster F2			
Item	*M*	*SD*	*M*	*SD*	*F*	*p*
The life I would live after my gambling is no longer problematic is vivid in my mind	6.00	0.78	4.47	1.46	10.02	0.004
The life I would live after my gambling is no longer problematic is vague in my mind	2.45	1.67	4.27	2.09	5.58	0.03
I long for the life I would live without problem gambling	6.09	1.22	4.47	1.69	7.35	0.01
When my gambling is no longer problematic, I will feel safe and secure in my life	6.09	0.83	4.67	1.72	6.40	0.02
It makes me feel anxious to think about the life I would live without gambling	3.45	2.30	4.40	1.72	1.44	0.24
The life I would live after I stop gambling problematically will be full of meaning	6.27	0.91	5.33	1.18	4.89	0.04
After my gambling is no longer problematic, I will feel more love in my life	5.82	1.25	4.53	1.73	4.38	0.05
Readiness to Change	7.27	2.15	6.40	2.44	0.89	0.35
Desire to Change	6.91	2.17	5.27	1.71	4.68	0.04

#### Behavior Change Measures

All participants were asked to rate the extent to which they felt ready to change their gambling behavior, in addition to the extent to which they desired change. Participants were also asked whether they had made a previous attempt to change their gambling behavior. Results indicated that participants in Cluster F1 (*M* = 7.27, *SD* = 2.15) did not differ from participants in Cluster F2 (*M* = 6.40, *SD* = 2.44) in the extent to which they were ready to change their gambling behavior, *F*(1,24) = 0.89, *p* = 0.35. However, participants in Cluster F1 (*M* = 6.91, *SD* = 2.17) reported a significantly greater desire to change their gambling behavior than did participants in Cluster F2 (*M* = 5.27, *SD* = 1.71), *F*(1,24) = 4.68, *p* = 0.04. Lastly, there were no cluster differences in the likelihood that a previous attempt to change their gambling behavior had been made, *X*^2^(1) = 0.54, *p* = 0.46.

## Discussion

The aim of the current study was to learn about how problem gamblers think and feel about their life before gambling became problematic, as well as their anticipated future when their gambling is no longer problematic. To this end, we classified concrete experiential accounts of the past as well as the future provided by problem gamblers. Doing so allowed for the possibility that there is more than one qualitatively distinct lived past experience or anticipated future experience. Results from the current study help to articulate the shared meaning that people give to their past lived experiences and the future they envision for themselves without problem gambling. Importantly, results also shed light on the conditions under which a past or future focus can effectively ready oneself for behavior change.

There were two different ways that gamblers wrote about their past experiences. The first (and most common) way to describe their past was of general positivity. These gamblers placed their past before gambling in an idealistic light, emphasizing the quality of their character, their relationships, and the array of meaningful activities they participated in. They also contrasted their favorable past against the hardships they currently face as a result of their gambling, and reported a longing to return to their past before gambling. The clustering of these constituents suggests that gamblers with a positive past may experience nostalgia as a result of the discontinuity that their problematic gambling behavior caused (Kim and Wohl, 2015; Sedikides et al., 2015; Salmon et al., 2018; Wohl et al., 2018). A second way that gamblers wrote about their past was of a life that was already quite difficult before gambling became problematic. The negative aspects of their past experiences involved periods of boredom or general dissatisfaction, as well as traumatic events that served as a trigger for the onset of their gambling. People who described such difficulties in their past also expressed (an unprompted) unwillingness to change their gambling behavior. As such, these people may be motivated to continue gambling to cope with the negative life events in their past (Blaszczynski and Nower, 2002; Stewart and Zack, 2008).

There were also two different ways that gamblers envisioned their future. The first way to describe their future was overwhelmingly positive. Specifically, people wrote optimistically about their possible future without problematic gambling, emphasizing that they will have a better financial situation and, as a result, will be able to engage in more meaningful activities and ultimately be happier. They also reported that this optimistic future is vivid in their mind. These findings are in line with prior research suggesting that optimism is associated with the ability to generate vivid mental imagery of positive future events (Blackwell et al., 2013; Ji et al., 2017). The second way that gamblers described their future was with ambivalence. Although participants were asked to envision a life when their gambling was no longer problematic, the hardships described in participants’ narratives were often centered on the process of quitting gambling or the uncertainty associated with what life may look like without gambling. Indeed, gamblers with ambivalence about their future reported that this future is vague in their mind. This may be due to the skewed temporal orientation commonly reported by disordered gamblers (Toneatto, 1999) in which their shorter time horizons prevent them from predicting events far into their future (Hodgins and Engel, 2002).

Importantly, the results of this numerically aided phenomenological study also suggest that each temporal orientation may be a source of motivation for self-directed change, but only when that temporal orientation is perceived to be positive. For example, gamblers with a positive past reported that they longed to return to this favorable time in their life. As a result, gamblers reported both a greater readiness and desire to change their gambling behavior than did gamblers with a difficult past. In line with gamblers’ experiential narratives, prior research has established the motivating properties of discontinuity-induced nostalgia (Kim and Wohl, 2015; Salmon et al., 2018; Wohl et al., 2018). Specifically, nostalgic reverie for the pre-addicted self heightens the extent to which people are ready for change (Kim and Wohl, 2015), in addition to increasing the likelihood that people will make an attempt to quit or cut down on an addictive behavior (i.e., gambling and drinking) over time (Salmon et al., 2018; Wohl et al., 2018). Therefore, a past focus that elicits nostalgia may have the greatest behavior change utility among gamblers who have a positive past they desire to reclaim.

Similarly, gamblers who anticipated a positive future also reported that they long for a future in which they are free from gambling problems, and reported a greater desire to change their gambling behavior than did gamblers who were ambivalent toward their future. However, there were no differences between clusters on the extent to which gamblers were ready to change their behavior. Importantly, gamblers who felt optimistic about their future without gambling also reported that this future is very vivid in their mind. As such, these gamblers may be able to use these vivid fantasies about their desired future to create a commitment to the goal of changing their gambling behavior (Oettingen et al., 2001). This mental contrasting of the desired future and current reality may lead people to take action to change their behavior (Oettingen, 2000). Moreover, having a positive outlook toward the future is predictive of motivation to attain a specific goal (i.e., self-directed change), though this may only be true for those who view changing their behavior as being instrumental to achieving their desired future (Van Calster et al., 1987). Therefore, a future focus that elicits vivid thoughts about a desired future may have the greatest behavior change utility among gamblers who are optimistic about the future they want to attain.

On the other hand, it is unlikely that a specific temporal orientation will facilitate self-directed change when that temporal orientation is perceived to have negative elements. For example, gamblers who described a difficult past before gambling reported fewer positive emotions associated with their lived past than did gamblers with a positive past. Moreover, these gamblers expressed an unwillingness to stop gambling, citing various reasons for continued play (e.g., the excitement). Gambling is often used as a maladaptive coping method to distract oneself from having to deal with the problems in their life (Gupta et al., 2004; Nower et al., 2004). In addition, gambling can also fill a void in one’s life, typically through alleviating boredom (Wood and Griffiths, 2007). Given that gambling may offer an escape to those with a difficult past, gamblers may not readily rely on a past focus when attempting to change their behavior. Rather, such gamblers may be more apt to draw on the promise of a brighter future focus, as gamblers can make a new life for themselves free from their past adversities.

In a like manner, gamblers who are ambivalent (i.e., they anticipate both positive and negative elements) toward their future reported that their future is vague and anticipated fewer positive emotions associated with this future than did gamblers who were optimistic. These gamblers also expressed a resistance toward changing their behavior, which may stem from the conflict between their readiness to change (i.e., acknowledging that change is in their best interest) and their desire for change. Indeed, resistance is often met with a reduced desire for change (Markland et al., 2005). Ambivalence may also be due to the fact that gamblers reported that they have difficulties envisioning a future beyond the process of quitting. Having a shortened time horizon makes the future difficult to plan for Hodgins and Engel (2002), as imagined futures often lack detail and contextual information (Noël et al., 2017). Among those who have difficulty imagining a future beyond the difficulties associated with changing their gambling behavior, a future focus is not likely to facilitate self-directed change. Instead, a past focus may serve as a vivid reminder of what life was like before their gambling became problematic and offer a clear image of what can be reclaimed through behavior change.

Indeed, the results of the current study suggest that gamblers may be less likely to draw on a specific temporal dimension as a source of behavioral motivation if such a period in time is not perceived to be positive. Yet to be explored is whether manipulating temporal focus may influence motivation to change among those with a difficult past or who feel ambivalent toward their future. For example, it is possible that gamblers with a difficult past may still reap the benefits of nostalgic reflection, but only when prompted to wax nostalgic about a time before the addictive behavior when they felt safe and secure. This may be accomplished by reminding the person living with addiction that nostalgia is a common human emotion experienced by everyone at some point in life (see Wildschut et al., 2006), that addictive behavior tends to ebb and flow, and thus to focus on a time when the addictive behavior was absent or not problematic. Similarly, gamblers who are ambivalent toward the future may also benefit from instructions that guide them toward generating vivid future imagery about what life will be like when their gambling problems are absent. Despite the difficulties many gamblers experience envisioning a future without gambling, being guided through the process of creating vivid possible future selves may motivate them to attain this desired future. Future research is encouraged to examine this possibility.

The results of the current study offer preliminary insight into the meaning that problem gamblers give to their lived past experiences before their gambling became problematic, as well as their imagined futures when their gambling will no longer be problematic. These insights are tentative due to the small sample size, however, findings do align with the extant literature on the behavior change utility of nostalgia as well as the literature on the desire for a better possible future as motivation for behavior change. As such, we have some confidence that our findings have basic and applied significance for understanding how to motivate behavior change among people living with addiction. Specifically, nostalgia appears to be an important factor in readying oneself for change when there is a readily accessible positive past to draw upon. From an applied perspective, treatment providers are encouraged to discuss with their clients how they perceive their lived past and anticipated future without gambling and use positive anchors (e.g., the quality of their character, relationships, or activities) in each temporal dimension to facilitate change. When one’s life before gambling is filled with distress, clients can be directed toward creating a more vivid future for themselves in which they will no longer gamble.

This study has a couple limitations that should be noted. First, due to the qualitative nature of this study, the sample size is quite small. Although the sample sizes were deemed appropriate for the numerically aided phenomenological assessment, the quantitative results reported should be interpreted with caution. The low sample size also prevents direct comparisons from being made between conditions on various outcome measures, such as the extent to which each temporal dimension is vivid in their mind. Rather, these comparisons are intended to be descriptive and provide further insight into the nature of each cluster. To be able to draw such conclusions, future research would do well to replicate and extend the outcomes associated with each temporal focus with sufficiently powered samples. Second, participants were assigned to either respond to writing prompts and follow-up items about their past before gambling or their future without gambling. Future research is encouraged to address this limitation by assessing participants’ natural dispositions to their past before gambling became problematic and their anticipated future without problematic gambling, as well as having participants complete full-scale measures of the outcomes of interest (e.g., longing for the past and future). Assessing gamblers’ natural temporal dispositions will also allow for the possibility that some people may be focused on both the past and future, while others may be focused on neither. Doing so would also provide a better understanding of how each temporal focus is associated with motivation to change addictive behavior, as well as their relative behavior change utility.

Although addiction is difficult to overcome, some people are motivated to take action to change their behavior. Longing for one’s life before the addictive behavior took hold or longing for a future when the addictive behavior will no longer be problematic is one such source of motivation. Importantly, using a numerically aided phenomenological approach, we demonstrated that the motivating properties of reflecting on the past and future are more pronounced when one’s life, character, relationships, and activities before gambling are perceived to be positive or when one’s future emotions, finances, and activities without gambling are expected to be positive. People engaging in addictive behaviors are encouraged to draw upon fond memories from the past as well as optimistic expectations for the future when gathering motivation to take action to change addictive behavior.

## Data Availability Statement

The raw data supporting the conclusions of this article will be made available by the authors, without undue reservation.

## Ethics Statement

The studies involving human participants were reviewed and approved by the Carleton University Research Ethics Board-B (CUREB-B). The patients/participants provided their written informed consent to participate in this study.

## Author Contributions

MS and MW worked together in the conception and design of this work. MS collected and analyzed the data, as well as drafting and revising the manuscript. MW provided substantial revisions to the manuscript. Both authors reviewed and approved the final version to be published.

## Conflict of Interest

The authors declare that the research was conducted in the absence of any commercial or financial relationships that could be construed as a potential conflict of interest.

## Footnotes

1 https://osf.io/nujya/?view_only=862d3b9ca76944c586223ccc962dcfe5
